# Diagnostic Performance of Generative Adversarial Network-Based Deep Learning Methods for Alzheimer’s Disease: A Systematic Review and Meta-Analysis

**DOI:** 10.3389/fnagi.2022.841696

**Published:** 2022-04-21

**Authors:** Changxing Qu, Yinxi Zou, Yingqiao Ma, Qin Chen, Jiawei Luo, Huiyong Fan, Zhiyun Jia, Qiyong Gong, Taolin Chen

**Affiliations:** ^1^Huaxi MR Research Center (HMRRC), Department of Radiology, West China Hospital, Sichuan University, Chengdu, China; ^2^State Key Laboratory of Oral Diseases, National Clinical Research Center for Oral Diseases, West China School of Stomatology, Sichuan University, Chengdu, China; ^3^West China School of Medicine, Sichuan University, Chengdu, China; ^4^Department of Neurology, West China Hospital, Sichuan University, Chengdu, China; ^5^West China Biomedical Big Data Center, West China Clinical Medical College of Sichuan University, Chengdu, China; ^6^College of Education Science, Bohai University, Jinzhou, China; ^7^Department of Radiology, West China Xiamen Hospital of Sichuan University, Xiamen, China; ^8^Research Unit of Psychoradiology, Chinese Academy of Medical Sciences, Chengdu, China; ^9^Functional and Molecular Imaging Key Laboratory of Sichuan Province, Department of Radiology, West China Hospital of Sichuan University, Chengdu, China

**Keywords:** generative adversarial networks (GANs), Alzheimer’s disease, mild cognitive impairment (MCI), diagnosis, psychoradiology, systematic review, meta-analysis

## Abstract

Alzheimer’s disease (AD) is the most common form of dementia. Currently, only symptomatic management is available, and early diagnosis and intervention are crucial for AD treatment. As a recent deep learning strategy, generative adversarial networks (GANs) are expected to benefit AD diagnosis, but their performance remains to be verified. This study provided a systematic review on the application of the GAN-based deep learning method in the diagnosis of AD and conducted a meta-analysis to evaluate its diagnostic performance. A search of the following electronic databases was performed by two researchers independently in August 2021: MEDLINE (PubMed), Cochrane Library, EMBASE, and Web of Science. The Quality Assessment of Diagnostic Accuracy Studies-2 (QUADAS-2) tool was applied to assess the quality of the included studies. The accuracy of the model applied in the diagnosis of AD was determined by calculating odds ratios (ORs) with 95% confidence intervals (CIs). A bivariate random-effects model was used to calculate the pooled sensitivity and specificity with their 95% CIs. Fourteen studies were included, 11 of which were included in the meta-analysis. The overall quality of the included studies was high according to the QUADAS-2 assessment. For the AD vs. cognitively normal (CN) classification, the GAN-based deep learning method exhibited better performance than the non-GAN method, with significantly higher accuracy (OR 1.425, 95% CI: 1.150–1.766, *P* = 0.001), pooled sensitivity (0.88 vs. 0.83), pooled specificity (0.93 vs. 0.89), and area under the curve (AUC) of the summary receiver operating characteristic curve (SROC) (0.96 vs. 0.93). For the progressing MCI (pMCI) vs. stable MCI (sMCI) classification, the GAN method exhibited no significant increase in the accuracy (OR 1.149, 95% CI: 0.878–1.505, *P* = 0.310) or the pooled sensitivity (0.66 vs. 0.66). The pooled specificity and AUC of the SROC in the GAN group were slightly higher than those in the non-GAN group (0.81 vs. 0.78 and 0.81 vs. 0.80, respectively). The present results suggested that the GAN-based deep learning method performed well in the task of AD vs. CN classification. However, the diagnostic performance of GAN in the task of pMCI vs. sMCI classification needs to be improved.

**Systematic Review Registration:** [PROSPERO], Identifier: [CRD42021275294].

## Introduction

Alzheimer’s disease (AD) is the most common form of dementia and is characterized by a progressive decline in memory and other cognitive functions. Notably, the pathophysiological processes of AD begin decades before clinical symptoms appear (Sperling et al., 2011; Atri, 2019; Matsuda et al., 2019); thus, early diagnosis and intervention are particularly important in AD management (Martí-Juan et al., 2020; Ansart et al., 2021). Mild cognitive impairment (MCI) is the prodromal stage, with symptoms occurring up to decades before dementia onset (Petersen, 2004; Misra et al., 2009). Approximately 10–15% of patients with MCI may progress to AD (pMCI) each year (Petersen et al., 2001), while the remaining patients may remain stable in the MCI stage (sMCI) (Li et al., 2016; Spasov et al., 2019). Studies examining the difference between AD and cognitively normal groups and between patients with pMCI and sMCI might facilitate the prediction of disease progression and help to provide the time window for administering potential disease-modifying therapy.

Neuroimaging biomarkers have been widely used in studies of AD to explain the underlying pathophysiological processes (McKhann et al., 2011; Chetelat, 2018). According to the National Institute on Aging and the Alzheimer’s Association (NIA-AA) research framework, biomarkers for the AD continuum were classified as AT(N) for amyloid, tau and neurodegeneration (Jack et al., 2016). A indicates amyloid-beta (Aβ) protein deposition, as reflected on amyloid positron emission tomography (PET) images (Jack et al., 2008). T indicates tau protein accumulation, as reflected by tau PET imaging (Cho et al., 2016). N indicates biomarkers of neurodegeneration or injury, including a reduction in glucose metabolism in the temporoparietal region, as reflected by fluorodeoxyglucose positron emission tomography (FDG-PET) imaging, and hippocampal atrophy observed using structural magnetic resonance imaging (MRI) (Jagust et al., 2007; Jack, 2011; Arbizu et al., 2018). The development of diagnostic methods based on these neuroimaging biomarkers is important to improve the diagnosis of AD, especially in the prodromal stage (Chetelat, 2018).

Artificial intelligence (AI) has been increasingly important in clinical diagnosis for the past few years. Psychoradiology with AI are emerging research directions for brain disorders (Lui et al., 2016). As one of the most important AI techniques, deep learning performs well in image processing for image detection, classification, and segmentation (Lee et al., 2017; Suzuki, 2017). It has been applied in some studies to achieve an accurate diagnosis of AD based on features extracted from AD-related images. Multiple deep learning models are being applied for the early detection and prediction of AD, such as convolutional neural networks (CNNs) (Zhou J. et al., 2021), autoencoders (AEs) (Ju et al., 2019), and deep belief networks (DBNs) (Shen et al., 2019; Lin E. et al., 2021).

The generative adversarial network (GAN) is a recent model first proposed by Goodfellow et al. (2014). It is a generative model mainly used for image processing based on the adversarial training of two components: the generative network (G) and the discriminative network (D). Fake images generated by this model, which highly resemble the real images, might exercise the same function as real images in disease diagnosis. In recent years, GAN has shown application value in diagnosing AD by providing image processing support, including quality improvement for low-dose PET images or 1.5-T MRIs (Wang et al., 2018; Ouyang et al., 2019; Zhou X. et al., 2021), predicting brain images at a future time point (Wegmayr et al., 2019; Zhao et al., 2021), data augmentation for network training (Islam and Zhang, 2020; Sajjad et al., 2021), and interconversion of PET and MRI data (Gao et al., 2021; Lin W. et al., 2021). With the GAN-based deep-learning classification framework, a more accurate diagnosis of AD is promising and may be achieved.

Some recent reviews reported the application of GAN in AD predictions and image classification. Logan et al. (2021) reported the application value of GAN in improving image quality and converting the modality. However, only two studies were included, and the results for the AD diagnosis were not reported. Lin E. et al. (2021) reported the application of GAN in a mouse model of AD with genomic data. Both studies were not comprehensive and did not include any data analysis for the AD diagnosis. To our knowledge, a gap exists in the meta-analysis for GAN application in the diagnosis of AD. This study systemically reviewed studies examining the application of GAN-based deep learning methods in the diagnosis of AD and subsequently performed a meta-analysis evaluating their diagnostic performance to fill this gap.

## Materials and Methods

This study was conducted according to the Preferred Reporting Items for a Systematic Review and Meta-analysis of Diagnostic Test Accuracy Studies (PRISMA-DTA) statement (McInnes et al., 2018).

### Protocol and Registration

This study was registered on PROSPERO with the registration number CRD42021275294.

### Focused Question

The focused question of this study is what is the performance of GAN in the diagnosis of AD?

### Patients, Intervention, Comparison, Outcome and Study Design Criteria

This study followed the Patients, Intervention, Comparison, Outcome and Study design (PICOS) criteria:

Patients (P): patients with AD or MCI.

Intervention (I): GAN-based deep learning methods for the diagnosis of AD. Specifically, the task of AD diagnosis referred to the AD vs. CN classification and pMCI vs. sMCI classification.

Comparison (C): the deep learning methods without GAN.

Outcome (O): the performance for the diagnosis of AD, including accuracy, sensitivity, specificity, and the area under the curve (AUC) of the summary receiver operating characteristic curve (SROC).

Study design (S): studies using neuroimaging data.

### Literature Search

A search of the following electronic databases was performed by two researchers (CQ and YZ) independently in August 2021: MEDLINE (PubMed), Cochrane Library, EMBASE, and Web of Science. The database coverage was up to August 2021. In addition, a manual search was conducted of references of the initially included articles and relevant reviews. The detailed search strategy is displayed in Supplementary Material.

### Inclusion and Exclusion Criteria

The inclusion and exclusion criteria followed the PICOS criteria.

Inclusion criteria: (1) participants who were clinically diagnosed with AD or MCI; (2) the application of GAN in the deep learning models; (3) report of the performance for diagnosis; and (4) diagnosis based on neuroimaging data (PET, MRI, etc.).

Exclusion criteria: (1) participants diagnosed with other brain disorders, such as brain tumors; (2) report of an assessment of generated image quality only, such as the peak signal to noise ratio (PSNR) and structural similarity (SSIM); (3) diagnosis based on other subjects rather than images; (4) conference abstracts (published abstracts of papers participating in academic conferences without the full text), editorials, letters, or review articles.

### Article Screening

Two researchers (CQ and YZ) independently performed the screen according to the PICOS criteria. The initial screen was performed by reading titles and abstracts. The full text was then read for further screening. A consensus was finally reached through negotiation in cases of any divergence between the two researchers.

### Data Extraction

A self-developed data extraction form was used by two researchers (CQ and YZ) independently. The following data were collected: author, year, country, data, participants, structure of the model, type of GAN, function of GAN, classification task, and performance.

### Quality Assessment

The quality of the included studies was assessed by two researchers independently with the Quality Assessment of Diagnostic Accuracy Studies-2 (QUADAS-2) tool. Assessment domains were as follows: risk of bias (patient selection, index test, reference standard, and flow and timing) and applicability concerns (patient selection, index test, and reference standard).

### Data Analysis

Stata 15 and MetaDiSc 1.4 software were used to analyze the data. For the accuracy analysis, researchers calculated the odds ratio (OR) with a 95% confidence interval (CI). Cochran’s *Q*-test and Higgins inconsistency index (*I*^2^)-test were performed to test heterogeneity. A fixed-effects model was used when non-significant heterogeneity was observed (*P* > 0.05 and *I*^2^ < 50%); otherwise (*P* < 0.05 or *I*^2^ > 50%), a random-effects model was applied.

The true positive (TP), false negative (FN), false-positive (FP), and true negative (TN) were calculated, and 2 × 2 tables were plotted based on the performance for diagnosis (accuracy, sensitivity, specificity, and other parameters) reported. Based on the data calculated above, researchers adopted a bivariate random-effects model to calculate the pooled sensitivity and specificity with their 95% CIs. An SROC curve was constructed, and the AUC was calculated. The Spearman correlation coefficient was obtained, and a value greater than 0.5 with *P* < 0.05 indicated the presence of threshold effects. Heterogeneity was assessed using the same method described for accuracy. The narrative analysis was adopted for the studies excluded from the meta-analysis.

According to the pooled sensitivity and specificity of neuroimaging biomarkers for diagnosis reported in some meta-analyses and rules for evaluating the AUC of classification models (Bloudek et al., 2011; Morris et al., 2016), we proposed that a method with great potential for clinical application should meet the following criteria: the pooled sensitivity or specificity was greater than 0.90 and the AUC was greater than 0.90.

## Results

### Study Selection

**FIGURE 1:**
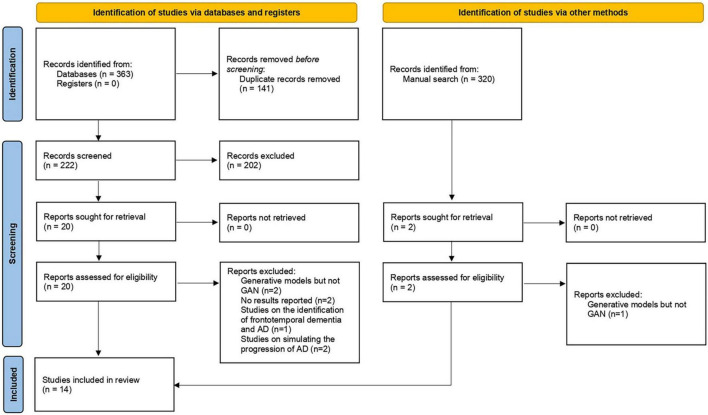
Flowchart of the study selection process (PRISMA flow chart).

In total, 364 articles were obtained by performing electronic and manual searches. Two hundred two articles were excluded during the initial screen, and 21 articles were selected after reading the full text. Eventually, 14 articles were included in this study. Researchers conducted a meta-analysis on 11 of these studies. The study selection process is displayed in Figure 1.

### Characteristics of the Included Studies

**FIGURE 2:**
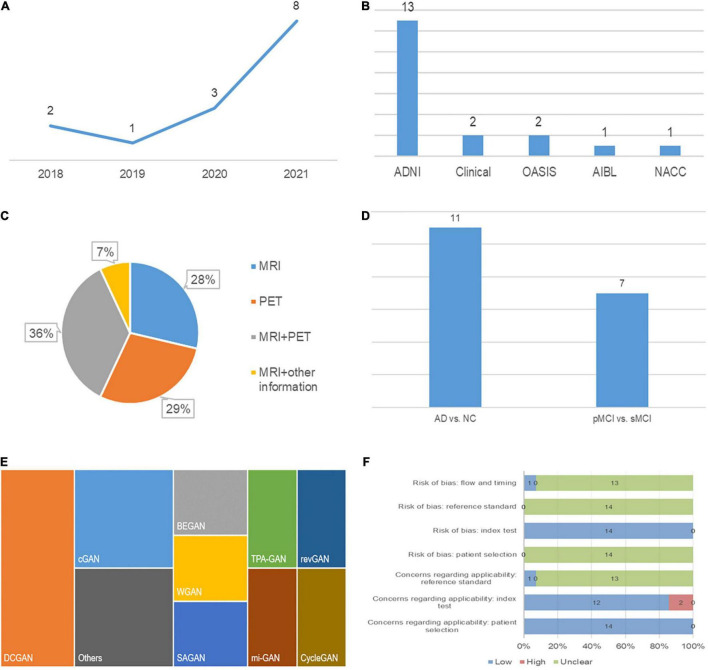
Characteristics of the included studies: **(A)** Publication year, **(B)** data source, **(C)** modality of data, **(D)** classification task, **(E)** type of GAN, and **(F)** quality assessment.

A detailed description of the study characteristics is provided in Table 1 and Figure 2. Regarding the publication year, all the included articles were published between 2018 and 2021, and more than half of them (8/14) were published in 2021 (Figure 2A; Baydargil et al., 2021; Gao et al., 2021; Han et al., 2021; Kang et al., 2021; Lin W. et al., 2021; Sajjad et al., 2021; Zhao et al., 2021; Zhou X. et al., 2021). Regarding the data source, neuroimaging data analyzed in 13 studies were mainly from the Alzheimer’s Disease Neuroimaging Initiative (ADNI) (Pan et al., 2018; Yan et al., 2018; Wegmayr et al., 2019; Islam and Zhang, 2020; Kim et al., 2020; Shin et al., 2020; Baydargil et al., 2021; Gao et al., 2021; Kang et al., 2021; Lin W. et al., 2021; Sajjad et al., 2021; Zhao et al., 2021; Zhou X. et al., 2021), and some data were from the Open Access Series of Imaging Studies (OASIS) (Han et al., 2021; Zhao et al., 2021), the Australian Imaging, Biomarker and Lifestyle Flagship Study of Aging (AIBL) and the National Alzheimer’s Coordinating Center (NACC) databases (Figure 2B; Zhou X. et al., 2021). Two studies established a test set from the collection of clinical data (Wegmayr et al., 2019; Kim et al., 2020). Regarding the data modality, 36 percent (5/14) of studies used data from two modalities (Figure 2C; Pan et al., 2018; Yan et al., 2018; Shin et al., 2020; Gao et al., 2021; Lin W. et al., 2021). One study used MRI and other clinical data (age, sex, education level, and other parameters) (Zhao et al., 2021). A deep convolutional GAN (DCGAN) was applied in 3 studies (Islam and Zhang, 2020; Kang et al., 2021; Sajjad et al., 2021), and conditional GAN (CGAN) was applied in 2 studies (Figure 2E; Yan et al., 2018; Shin et al., 2020). The type of GAN in the remaining studies varied. For the diagnostic task, 11 studies focused on the AD vs. CN classification (Pan et al., 2018; Islam and Zhang, 2020; Kim et al., 2020; Shin et al., 2020; Baydargil et al., 2021; Gao et al., 2021; Han et al., 2021; Kang et al., 2021; Lin W. et al., 2021; Sajjad et al., 2021; Zhou X. et al., 2021), and 7 studies were devoted to the pMCI vs. sMCI classification (Figure 2D; Pan et al., 2018; Yan et al., 2018; Wegmayr et al., 2019; Gao et al., 2021; Kang et al., 2021; Lin W. et al., 2021; Zhao et al., 2021). For the assessment of the diagnostic performance, accuracy was reported in all studies, while sensitivity and specificity were reported in 6 studies examining the AD vs. CN classification (Pan et al., 2018; Kim et al., 2020; Gao et al., 2021; Kang et al., 2021; Lin W. et al., 2021; Zhou X. et al., 2021) and 4 studies examining the pMCI vs. sMCI classification (Pan et al., 2018; Gao et al., 2021; Kang et al., 2021; Lin W. et al., 2021).

**TABLE 1:** Characteristics of the included studies.

Authors	Year	Country	Data		Participants					Structure of the model	Type of GAN	Function of GAN	Task of classification	Performance						
			Source	Modality	AD	MCI	pMCI	sMCI	CN					Accuracy	Sensitivity	Specificity	F1-score	Recall	Precision	AUC
Pan et al. (2018)	2018	China	ADNI^a^	MRI+PET	358	−	205	465	429	Two-stage: GAN+ LM3IL^b^	cycleGAN	Modality conversion	AD vs. CN; pMCI vs. sMCI	0.92; 0.79	0.90; 0.55	0.94; 0.83	0.91; 0.41	−	−	0.96; 0.76
Islam and Zhang (2020)	2020	United States	ADNI	PET	98	−	−	−	105	Two-stage: GAN+CNN^c^	DCGAN^d^	Data augmentation	AD vs. CN	0.71	−	−	−	−	−	−
Kim et al. (2020)	2020	Korea	ADNI; clinical	PET	139	−	−	−	347	Two-stage: GAN+SVM^e^	BEGAN^f^	Feature extraction	AD vs. CN	0.94	0.92	0.97	−	−	−	0.98
Wegmayr et al. (2019)	2019	Switzerland	ADNI; clinical	MRI	−	−	89	116	−	Two-stage: GAN+CNN	WGAN^g^	Aging simulation	pMCI vs. sMCI	0.73	−	−	0.71	0.75	0.68	−
Yan et al. (2018)	2018	United Kingdom	ADNI	MRI+PET	−	−	58	50	−	Two-stage: GAN+ Resnet	cGAN^h^	Modality conversion	pMCI vs. sMCI	0.82	−	−	−	−	−	0.81
Baydargil et al. (2021)	2021	Korea	ADNI	PET	25	−	−	−	148	GAN only	GAN	Anomaly detection	AD vs. CN	−	−	−	−	−	−	0.75
Gao et al. (2021)	2021	China	ADNI	MRI+PET	352	−	234	342	427	Two-stage: GAN+ DCN^i^	TPA-GAN^j^	Modality conversion	AD vs. CN; pMCI vs. sMCI	0.93; 0.75	0.92; 0.71	0.94; 0.78	0.92; 0.70	−	−	0.96; 0.78
Han et al. (2021)	2021	Japan	OASIS^k^	MRI	96	152	−	−	576	GAN only	SAGAN^l^	Anomaly detection	AD vs. CN	−	−	−	−	−	−	0.89
Kang et al. (2021)	2021	China	ADNI	MRI	187	−	138	181	229	Ensemble learning: discriminator of GAN+VGG16+ ResNet50	DCGAN	Transfer learning	AD vs. CN; pMCI vs. sMCI	0.90; 0.63	0.94; 0.58	0.84; 0.64	−	−	−	0.90; 0.62
Lin W. et al. (2021)	2021	China	ADNI	MRI+PET	362	−	183	233	308	Two-stage: GAN+CNN	revGAN^m^	Modality conversion	AD vs. CN; pMCI vs. sMCI	0.89; 0.71	0.90; 0.74	0.88; 0.68	−	−	−	0.88; 0.74
Sajjad et al. (2021)	2021	Pakistan	ADNI	PET	30	−	−	−	42	Two-stage: GAN+VGG16	DCGAN	Data augmentation	AD vs. CN	0.83	−	−	0.88	0.86	0.91	−
Zhou X. et al. (2021)	2021	United States	ADNI; AIBL^n^; NACC^o^	MRI	411	−	−	−	678	Two-stage: GAN+FCN^p^	GAN	Quality improvement	AD vs. CN	0.82	0.74	0.89	0.79	−	−	−
Shin et al. (2020)	2020	Korea	ADNI	MRI+PET	162	675	−	−	428	GAN only	cGAN	Modality conversion; classification	AD vs. CN	0.85	−	−	−	0.84	0.84	−
Zhao et al. (2021)	2021	China	ADNI; OASIS	MRI+other information	151	341	−	−	113	Two-stage: GAN+DenseNet	mi-GAN^q^	Aging simulation	pMCI vs. sMCI	0.78	−	−	0.74	0.71	0.78	−

*^a^Alzheimer’s Disease Neuroimaging Initiative;*

*^b^andmark-based Multimodal Multi-Instance Learning;*

*^c^Convolutional Neural Network;*

*^d^Deep Convolutional Generative Adversarial Network;*

*^e^ Support Vector Machine;*

*^f^Boundary Equilibrium Generative Adversarial Network;*

*^g^Wasserstein Generative Adversarial Network;*

*^h^Conditional Generative Adversarial Network;*

*^i^Dense Convolution Network;*

*^j^Task-induced Pyramid and Attention Generative Adversarial Network;*

*^k^Open Access Series of Imaging Studies;*

*^l^Self Attention Generative Adversarial Network;*

*^m^Reversible Generative Adversarial Network;*

*^n^Australian Imaging, Biomarker and Lifestyle Flagship Study of Aging;*

*^o^National Alzheimer’s Coordinating Center;*

*^p^Fully Convolutional Network;*

*^q^Multi-information Generative Adversarial Network.*

Regarding the function of image processing, one study applied GAN to generate higher-quality MRI data. Two studies stimulated the process of brain aging observed in MRI images (Wegmayr et al., 2019; Zhao et al., 2021). Two studies used GAN to augment imaging data and improve the training effects of the classifiers (Islam and Zhang, 2020; Sajjad et al., 2021). Five studies achieved conversion between PET and MRI data to provide supplementary data (Pan et al., 2018; Yan et al., 2018; Shin et al., 2020; Gao et al., 2021; Lin W. et al., 2021).

### Generative Adversarial Networks

GAN is composed of a G and a D. The goal of the GAN is to generate the image most similar to the real image through G-D competitions. As a random vector input, G generates a fake image. The goal of G is to make it as close as possible to the real image. As the generated and corresponding real image input, D provides a probability for the generated image being real (1 indicates real and 0 indicates fake). The goal of D is to identify fake images as accurately as possible. With the continuous adversarial training on G and D, the similarity between the image generated by G and the real image is maximized, and concurrently, the accuracy of D in identifying fake images is maximized. When G and D reach a Nash equilibrium state through training (the probability output by D is 1/2 each time), the model reaches the optimum. At this time, GAN outputs an image closest to the real image.

**FIGURE 3:**
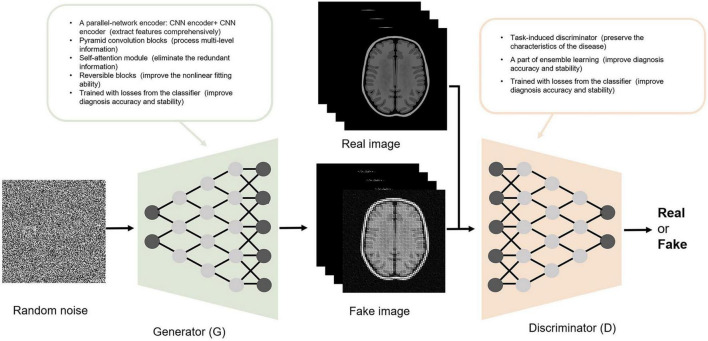
The structure of the GAN and some improvements reported in the included studies.

Except for the function of image processing, GANs have high structural flexibility. This property allows any differential function to be applied in G and D construction and cooperates with other recognized deep learning networks (such as CNN) to constitute the deep generative model. Yan et al. (2018) and Zhao et al. (2021) built the G based on U-net, and Yan et al. (2018) and Lin W. et al. (2021) established the Markovian-based D (PatchGAN). Additionally, the GAN framework embraces all types of loss functions and constraints, which provides individualized methods according to different tasks. The modified GAN was applied in the included studies and contributed to an improved diagnosis of AD. Some improvements in the structure of GAN and their contributions are shown in Figure 3. For the generator, Baydargil et al. (2021) proposed a parallel structure, with CNN extracting local features and DCN extracting global features. The generator produces images that are close to the real images using comprehensive features. Gao et al. (2021) and Han et al. (2021) added the self-attention module to focus the attention of the algorithm on specific regions instead of focusing indiscriminately on the whole image, reducing redundant information extraction. For the discriminator, Gao et al. (2021) added the task-induced mechanism. The task-induced discriminator focused not only on the quality of the generated images but also on whether AD pathological information was retained. In addition, the results of the downstream classification task were fed back to the generator and discriminator during training in the study by Zhou X. et al. (2021). This training may ensure the classification performance of the generated images.

### Quality Assessment

The quality assessment is described in detail in Figure 2F. Two studies had high concerns regarding the applicability (Baydargil et al., 2021; Han et al., 2021). In these studies, GAN was developed for anomaly detection, which is a screen for AD, while diagnosis is the main focus of our study. Applicability concerns of reference standard were low in the study by Han et al. (2021) because they clearly indicate that the diagnostic criteria for AD were the clinical dementia rating (CDR). In addition, the risk of bias in flow and timing was low in this study, as the authors ensured that the interval between CDR and MRI acquisition was as short as possible (Han et al., 2021).

### Diagnostic Performance of Generative Adversarial Network-Based Deep Learning Methods

#### The Task of Alzheimer’s Disease vs. Cognitively Normal Classification

**FIGURE 4:**
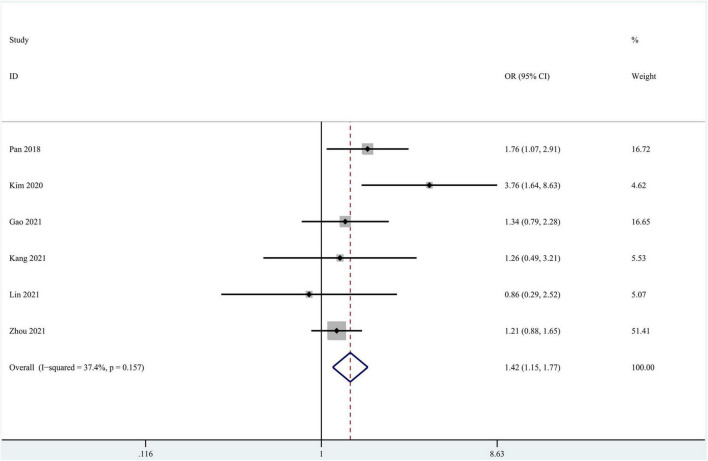
Forest plot of the accuracy in the task of AD vs. CN classification.

Eleven studies focused on the application of GAN to the task of AD vs. CN classification (Pan et al., 2018; Islam and Zhang, 2020; Kim et al., 2020; Shin et al., 2020; Baydargil et al., 2021; Gao et al., 2021; Han et al., 2021; Kang et al., 2021; Lin W. et al., 2021; Sajjad et al., 2021; Zhou X. et al., 2021). Meta-analyses were performed on 6 studies reporting the accuracy, sensitivity, and specificity (Pan et al., 2018; Kim et al., 2020; Gao et al., 2021; Kang et al., 2021; Lin W. et al., 2021; Zhou X. et al., 2021). The results of the meta-analyses are shown in Table 2. For the accuracy assessment, the pooled OR was 1.425 (95% CI: 1.150–1.766; *P* = 0.001). Heterogeneity among the studies was not significant (*I*^2^ = 37.4, *P* = 0.157), and the fixed-effects model was applied. This result revealed that GAN-based deep learning methods efficiently increased the accuracy of the task of AD vs. CN classification (Figure 4).

**TABLE 2:** The results of meta-analyses of the diagnosis of AD.

Task	Method	OR^a^ of accuracy	SEN^b^	SPE^c^	AUC^d^ of the SROC^e^	Spearman correlation coefficient
AD vs. NC	w/^f^ GAN	1.425* (1.150–1.766)	0.88 (0.82–0.93)	0.93 (0.90–0.95)	0.96 (0.94–0.97)	−0.029
	w/o^g^ GAN		0.83 (0.76–0.88)	0.89 (0.86–0.92)	0.93 (0.90–0.95)	0.257
pMCI vs. sMCI	w/GAN	1.149 (0.878–1.505)	0.66 (0.57, 0.75)	0.81 (0.76, 0.85)	0.81 (0.72–0.89)	1.000*
	w/o GAN		0.66 (0.57, 0.75)	0.78 (0.74, 0.82)	0.80 (0.74–0.87)	1.000*

*Statistically significant, p≤ 0.05. ^a^Odds ratio; ^b^sensitivity; ^c^specificity; ^d^area under the curve; ^e^summary receiver operating characteristic curve; ^f^ with; ^g^without.

**FIGURE 5:**
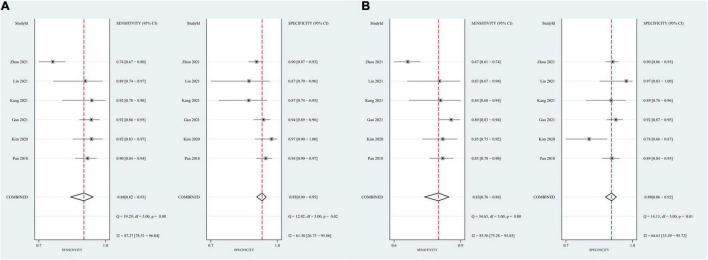
Forest plots showing the pooled sensitivity and specificity in the task of AD vs. CN classification. **(A)** The pooled sensitivity and specificity in the GAN group; **(B)** the pooled sensitivity and specificity in the non-GAN group.

**FIGURE 6:**
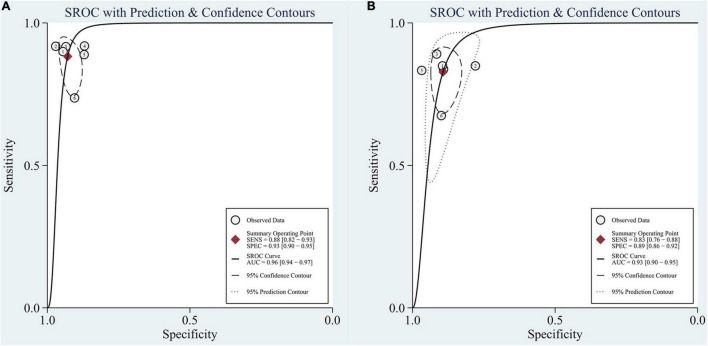
SROC curve for the task of AD vs. CN classification: **(A)** SROC curve for the GAN group and **(B)** SROC curve for the non-GAN group.

In the group with GAN, the pooled sensitivity was 0.88 (95% CI: 0.82–0.93), the pooled specificity was 0.93 (95% CI: 0.90–0.95), and the AUC of the SROC was 0.96 (95% CI: 0.94–0.97). Significant heterogeneity was observed in both sensitivity (*I*^2^ = 87.27, *P* = 0) and specificity (*I*^2^ = 61.30, *P* = 0.02). These values were much higher than those in the group without GAN (Figures 5, 6). Threshold effects were absent in both groups according to Spearman’s correlation coefficients (−0.029, *P* = 0.957; 0.257, *P* = 0.623). Generally, GAN-based deep learning methods were superior to the method without GAN and had great potential for clinical application based on the criteria described above.

The advantage of GAN was also observed in studies not included in the meta-analysis. Baydargil et al. (2021) reported that the AUC for the GAN-based method was 0.7, which was significantly higher than that of other methods. Han et al. (2021) reported the medical anomaly detection GAN (MAGAN) with an AUC of 0.89. Three studies showed higher accuracy of GAN-based methods (0.71, 0.85, and 0.83) (Islam and Zhang, 2020; Shin et al., 2020; Sajjad et al., 2021).

#### The Task of Progressing MCI vs. Stable MCI Classification

**FIGURE 7:**
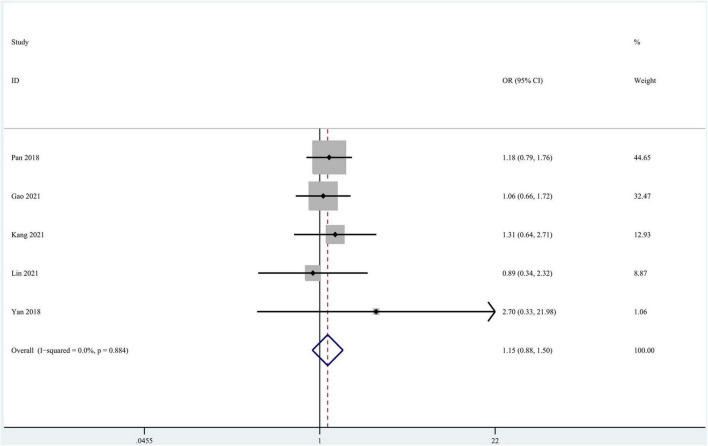
Forest plot of the accuracy in the pMCI vs. sMCI classification task.

Seven studies focused on the application of GAN to the task of pMCI vs. sMCI classification (Pan et al., 2018; Yan et al., 2018; Wegmayr et al., 2019; Gao et al., 2021; Kang et al., 2021; Lin W. et al., 2021; Zhao et al., 2021). A meta-analysis was performed on 5 studies reporting the accuracy (Pan et al., 2018; Yan et al., 2018; Gao et al., 2021; Kang et al., 2021; Lin W. et al., 2021), and another was performed on 3 studies reporting sensitivity and specificity (Pan et al., 2018; Gao et al., 2021; Lin W. et al., 2021). For accuracy, the pooled OR was 1.149 (95% CI: 0.878–1.505; *P* = 0.310). Heterogeneity among the studies was not significant (*I*^2^ = 0, *P* = 0.884), and the fixed-effects model was applied (Figure 7).

**FIGURE 8:**
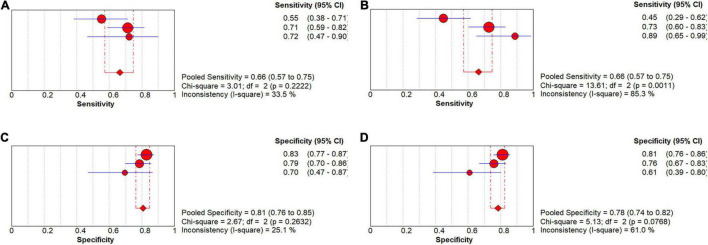
Forest plots showing the pooled sensitivity and specificity in the task of pMCI vs. sMCI classification. **(A)** The pooled sensitivity in the GAN group and **(B)** the non-GAN group. **(C)** The pooled specificity in the GAN group and **(D)** the non-GAN group.

**FIGURE 9:**
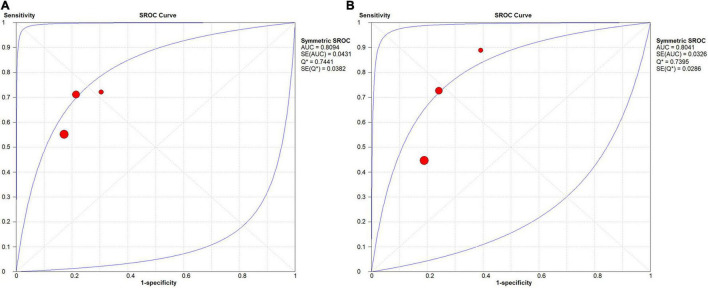
SROC curves for the task of pMCI vs. sMCI classification: **(A)** SROC curve for the GAN group and **(B)** SROC curve for the non-GAN group.

In the group with GAN, the pooled sensitivity was 0.66 (95% CI: 0.57–0.75), the pooled specificity was 0.81 (95% CI: 0.76–0.85), and the AUC of the SROC was 0.81 (95% CI: 0.72–0.89) (Figure 8). Low heterogeneity was observed in both sensitivity (*I*^2^ = 33.50, *P* = 0.22) and specificity (*I*^2^ = 25.10, *P* = 0.26). The specificity and AUC of the SROC were slightly higher than those in the group without GAN (Figure 9). Threshold effects were strong on both groups according to Spearman’s correlation coefficients. Overall, in the task of pMCI vs. sMCI classification, the differences between the group with GAN and the group without GAN were not significant.

## Discussion

In this study, we analyzed the performance of GAN in diagnosing AD. GAN-based deep learning methods significantly increased the accuracy, sensitivity, and specificity in the task of AD vs. CN classification. However, their diagnostic performance in the task of pMCI vs. sMCI classification was not remarkable.

### Performance of Generative Adversarial Network-Based Deep Learning Methods in the Task of Alzheimer’s Disease vs. Cognitively Normal Classification

Developments in disease-modifying therapy for AD have slowly progressed, with a failure rate of 99.6% in clinical trials (Cummings et al., 2014; Marinescu et al., 2019). Based on this information, identifying patients with early AD has become a focus in current studies (Chong and Sahadevan, 2005; Davis et al., 2018). Effective discrimination between AD and CN might help identify patients with AD in a timely manner and implement targeted interventions to delay disease progression.

Our study showed that GAN-based deep learning methods with different data modalities and different structures of the model all showed good performance in the task of AD vs. CN classification.

Regarding the data modality, some studies used MRI data, while others used PET data. Zhou X. et al. (2021) developed a GAN model to generate 3-T MRI scans from 1.5-T scans. Then, researchers trained a fully convolutional network (FCN) using generated 3-T MRI as inputs to complete the task of AD vs. CN classification. The classification efficiency was ensured by the concurrent training of the GAN and FCN. In that study, the FCN trained on the generated 3-T MRI data performed better than that trained on 1.5-T MRI data, with higher accuracy (0.84 vs. 0.82), sensitivity (0.74 vs. 0.67), and specificity (0.9037 vs. 0.8989). Sajjad et al. (2021) trained a VGG16 classifier on DCGAN-amplified PET data. Good performance of this classifier was reported in the task of AD vs. CN classification (accuracy: 0.83; recall: 0.86; precision: 0.91; F1-score: 0.88). Islam and Zhang (2020) reported an accuracy of 71.45% when using GAN-augmented PET data in the AD vs. CN classification, a 10% increase compared to the classifier trained on data without GAN augmentation.

Regarding the structure of the model, some researchers constructed anomaly detection models based on GAN to identify patients with AD. Baydargil et al. (2021) established a deep-learning model based on adversarial training for diagnosing AD. The G was an encoder-decoder network with the encoder a parallel feature extractor consisting of CNN and DCN, which were used for extracting local and global features from the real PET images, respectively. The G reconstructed a PET image based on these feature vectors and then input it to the encoder-type D for AD diagnosis. This study finally reported that the AUC of this method was 0.75. Han et al. (2021) proposed a medical anomaly detection GAN (MADGAN) using multiple adjacent brain MRI slice reconstruction to detect patients with AD by considering that AD is composed of the accumulation of subtle anomalies (AUC = 0.89).

Moreover, some researchers trained the classifier based on features extracted or images processed using GAN to complete the task of pMCI vs. sMCI classification. Kim et al. (2020) extracted features of two brain PET slices with the encoder-decoder D in GAN and trained an SVM classifier on these features to achieve accurate classification of AD and CN. Compared with the 2D-CNN model, the SVM classifier exhibited a 12.77% increase in accuracy, a 6.82% increase in sensitivity, and a 19.37% increase in specificity. Shin et al. (2020) constructed an end-to-end network based on the GAN model, with G for MRI-PET conversion and D for AD classification. The structure of this network is different from the conventional two-step structure, which starts from PET data generation based on MRI data to AD diagnosis with the generated PET data, leading to 0.85 accuracy and 0.84 precision and recall in the task of AD vs. CN classification.

All included studies used data of AD patients through clinical diagnosis rather than neuropathological examination. Although neuropathological diagnosis at autopsy serves as the gold standard for diagnosing AD (Hyman et al., 2012), data of AD diagnosed through it are sparse and difficult to obtain. Researchers may consider that small data sizes could limit the adequate training of deep learning networks and chose to use data of clinically diagnosed AD from large publicly available databases, such as ADNI, OASIS, AIBL, and so on. However, clinical diagnosis may be less accurate compared to neuropathological examination currently. One study reported that sensitivity for AD clinical diagnosis based on the NINCDS-ADRDA guidelines ranged from 70.9 to 87.3% and specificity ranged from 44.3 to 70.8% compared to the golden standard (Beach et al., 2012). This could affect the evaluation of the diagnostic performance of GAN-based deep learning methods.

### Performance of Generative Adversarial Network-Based Deep Learning Methods in the Task of Progressing MCI vs. Stable MCI Classification

MCI is a transition between CN and AD (Petersen, 2004). Patients with MCI who progress to AD are classified as having pMCI, while those who maintain stable disease conditions and even return to normal are classified as having sMCI (Petersen et al., 2001; Li et al., 2016). Efficient discrimination between pMCI and sMCI groups is beneficial for the early identification of patients at high risk of developing AD and helps further detect the high-risk factors responsible for disease progression. Using this approach, corresponding interventions might be scheduled, in turn delaying disease progression and decreasing the occurrence of AD.

In our study, GAN-based deep learning methods showed no remarkable classification performance in the task of pMCI vs. sMCI classification compared to the task of AD vs. CN classification. This difference is mainly attributed to the subtle pathological differences between patients with pMCI and sMCI (Kang et al., 2021). Compared to CN patients, significant hippocampal atrophy has been observed in both patients with pMCI and sMCI (Zeng et al., 2021). In this setting, the result is generally negative if the deep learning model only uses the whole hippocampal volume as the input feature in the task of pMCI vs. sMCI classification. A recent cohort study revealed that the volume of the bilateral subiculum and molecular layer in patients with pMCI was smaller than that in patients with sMCI, along with more rapid atrophy (Zeng et al., 2021). The volume of the hippocampal subregion is the main source of the difference between these two types. However, the volume of these subregions is very small, especially in 2D images. The difference in volume is difficult to capture using the deep learning method due to the floor effect and might provide an interpretation of the lack of remarkable performance in the task of pMCI vs. sMCI classification.

The included studies have attempted to overcome this limitation and achieve better performance in the task of pMCI vs. sMCI classification.

Some studies applied multimodal data to improve the performance. Lin W. et al. (2021) first developed a GAN with reversible blocks to achieve PET-MRI conversion. Then, they trained a 3D-CNN classifier (4 layers) by generating images of the hippocampus using these 2 modalities (PET and MRI) to complete the tasks of AD vs. CN and pMCI vs. sMCI classification. In this study, the hippocampus was set as the region of interest (ROI), which decreased unnecessary calculations and contributed to 89.05% accuracy in the AD vs. CN classification and 71.23% accuracy in the pMCI vs. sMCI classification. Gao et al. (2021) proposed a DCN classifier trained on MRI data and the corresponding PET data by GAN conversion. Pathwise transfer blocks were adopted to allow information communication across two paths of PET and MRI data. This approach enabled the classifier to make full use of complementary information of these images and improve the classification performance. Researchers performed a comparative analysis with the method without GAN and found that the GAN-based model exhibited better performance in both AD vs. CN and pMCI vs. sMCI classification tasks. Pan et al. (2018) and Yan et al. (2018) also used GAN to perform MRI-PET data conversion to compensate for insufficient training due to missing PET data. With classifiers trained on MRI data and the generated PET data, Yan et al. (2018) obtained a 7% increase in classification accuracy compared to the classifier trained on PET data with traditional augmentation. Pan et al. (2018) also reported better performance in both AD vs. CN and pMCI vs. sMCI classification tasks.

Some researchers applied the ensemble learning strategy to increase the accuracy and stability in the pMCI vs. sMCI classification task. Kang et al. (2021) devised a multimodal ensemble learning model for AD diagnosis based on three classifiers (GAN-D, VGG16, and ResNet50) trained on 11 MRI slices with the best diagnostic performance selected by the VGG16 classifier. The introduction of multiple slices and the multimodal classifier increased the accuracy and stability of the ensemble learning model in classification. Their result showed a 5.8% increase in accuracy of the ensemble learning model in the AD vs. CN classification compared to the single VGG16 classifier. Meanwhile, the three classifiers were separately analyzed, and the GAN-D was reported to be superior to VGG16 and ResNet50 classifiers in both AD vs. CN and pMCI vs. sMCI classification tasks, indicating the advantage of GAN to some extent. The differences in pathological changes between patients with pMCI and sMCI increases with aging. Based on this information, some studies simulated the process of aging observed in MRI data to predict disease progression. Zhao et al. (2021) constructed a 3D patch-based multi-information GAN (MI-GAN) model to generate aging-related MRI images based on baseline MRI image data and related clinical information. Then, they trained a 3D Multi-Classification Model on these aging images to perform the pMCI vs. sMCI classification. The results showed 78.45% accuracy, a 3.01% increase compared to the deep neural networks and ensemble learning models. Wegmayr et al. (2019) also simulated the aging process of patients (as evidenced by MRI data) using time as the variable and then established a pMCI-sMCI classifier trained on aging images to identify patients at high risk of developing AD. The authors found that the classifier trained on aging images displayed a higher accuracy (0.73 vs. 0.70) and F1-score (0.71 vs. 0.61) than the classifier trained on baseline images.

### The Function of Generative Adversarial Network in the Diagnostic Model

The excellent performance of GAN-based deep learning methods in diagnosing AD is attributed to the powerful functions of image processing by GAN and the model structure. In most of the included studies, the diagnostic model included 2 stage: the first was image processing by GAN and the second was the classifier established with other algorithms (primarily CNN) and training on images processed in stage one. Stage 1, instead of stage 2, is recognized as the critical stage for good performance in diagnosing AD (Sabuncu and Konukoglu, 2015). Therefore, the function of GAN determines the final effects of the entire diagnostic model. In the GAN, D provides a self-adaptive loss function based on different tasks and data, which is known as GAN-loss for G. The GAN-loss function might become powerful with the discriminative ability of D strengthening during training. This powerful loss function might promote image processing by G. In contrast, in other generative models, the image processing ability is limited, as their training is confined to the loss function preset. GAN, therefore, might provide images of higher quality for the diagnostic model and increase the diagnostic performance.

**FIGURE 10:**
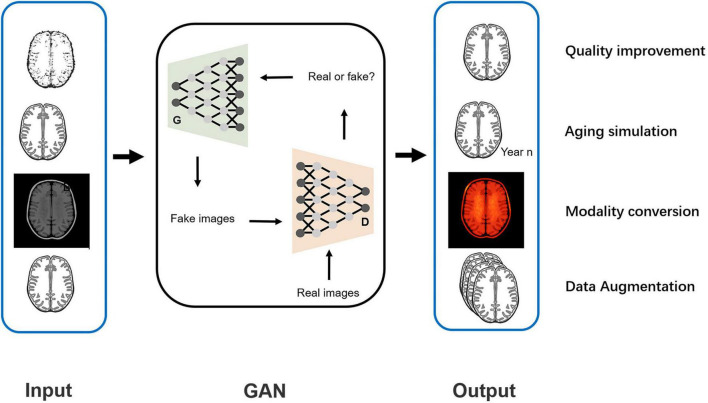
Schematic diagram of the function of image processing using GAN.

Specifically, the GAN provided image processing from four aspects in the included studies: quality improvement, aging simulation, data augmentation, and modality conversion (shown in Figure 10). The next section provides a description of the four functions and their effects on the AD diagnosis.

#### Quality Improvement

A GAN generates MRI data at high magnetic field strength from data collected at a low strength. Zhou X. et al. (2021) constructed a diagnostic model for AD based on 3-T MRI data generated by GAN, whose image quality was significantly higher than that of 1.5-T MRI scans based on SNR, BRISQUE, and NIQE metrics. The clear presentation of the diagnostic features by improving quality is the cause for the excellent performance of this deep learning method in disease diagnosis. Hippocampal atrophy on MRI is considered as potential neuroimaging markers for neurodegeneration in patients with AD. It might be presented much more clearly with a more accurate segmentation boundary in 3-T MRI than in 1.5-T MRI (Ho et al., 2010). A study also reported a much more widespread pattern of significant atrophy in the temporal lobe when scanned at 3-T vs. 1.5-T in the AD vs. CN classification. Due to the quality improvement function of GAN, the classifier easily obtained more accurate diagnostic features and detected differences between the AD and CN cohorts in these target areas, contributing to better classification performance.

Moreover, the increase in the quality of low-dose PET images obtained using GAN was reported in some studies. Wang et al. (2018) obtained full-dose PET images from low-dose images using the CGAN with a 3D U-net-like generator. The skip connections strategy was applied to combine hierarchical features. The authors obtained imaging data from healthy subjects and patients with MCI with the highest PSNR and the lowest NMSE compared to the methods based on the sparse representation and CNN. Additionally, the difference in the SUV between the PET images generated using GAN and the real full-dose PET images was the smallest. Ouyang et al. (2019) added an amyloid status classifier to GAN to ensure the preservation of pathological features in the generated image, which was superior to the CNN-based method, with a 1.87 dB PSNR, 2.04% SSIM, and 24.75% RMSE. The reductions in glucose metabolism in the parietal lobe, posterior cingulate, and temporal regions observed using FDG-PET are known as potential biomarkers reflecting the pathophysiological process of neuronal degeneration and injury in patients with AD (Zhang et al., 2011). High-dose PET images contain less noise than low-dose images, preserving more details of these diagnostic regions and more disease features that could be used in the classification. This finding also supports the good diagnostic performance of GAN-based deep learning methods.

#### Aging Simulation

In some studies, GAN was applied to predict disease progression by simulating cerebral aging with time as the variable. Zhao et al. (2021) generated aging MRI data based on baseline MRI scans and other clinical information. The generated images at year 1 and year 4 were highly similar to the real images (SSIM: 0.945 ± 0.038, 0.943 ± 0.028). Wegmayr et al. (2019) also built a model to simulate cerebral aging based on WGAN. These generated aging images had the same role as real images in the pMCI vs. sMCI classification (accuracy: 0.73, F1-score: 0.71). Some longitudinal pathological changes were observed using the aging simulation. In patients with pMCI, atrophy of the temporal lobe may extend forward to the parietal lobe, frontal lobe, lateral occipital cortex, and subsequent anterior cingulate cortex during the aging process (McDonald et al., 2009). Meanwhile, losses in the hippocampal and whole-brain volumes along with increasing ventricular volume have been reported (Jack et al., 2005; Hu et al., 2014). The larger differences between patients with pMCI and sMCI observed in the aging images compared to those observed in baseline images were shown, and the classifier performed better in the classification. The aging simulation function of GAN contributes to excellent performance in the task of pMCI vs. sMCI classification.

#### Data Augmentation

Large datasets with labels are commonly the basis of the construction and training of deep learning frameworks, especially for supervised learning. However, the medical images are labeled largely based on the subjective experience and professional level of experts and might be affected by the image quality. Notably, labeling images from patients with different stages of AD is more challenging. Sparse labeled medical images might limit the application of deep learning in the diagnosis of AD (Tajbakhsh et al., 2020). The GAN can compensate for data insufficiency during the development of AD-related deep learning frameworks through the augmentation of PET and MRI data. Sajjad et al. (2021) performed augmentation on PET data with the DCGAN model. They reported high levels of PSNR (0.82, 0.73) and SSIM (25.66, 22.85) in generated images for patients with AD and MCI. Additionally, the corresponding classifier exhibited good performance in the task of AD vs. CN classification with an accuracy of 0.78. Islam and Zhang (2020) reported mean PSNR and SSIM values of 32.83 and 77.48, respectively, on the generated PET image data. The accuracy of the classifier based on these augmented data was 71.45% in the AD vs. CN classification, which was evidently higher than the value of 10% obtained using the classifier without data augmentation.

#### Modality Conversion

The type of data is also a vital factor contributing to diagnostic performance. In our study, 36% (5/14) of the included studies used multimodal data (PET and MRI) for analysis. PET data commonly provide metabolic information that is helpful in determining the diagnosis. For example, the reduction in glucose metabolism in the bilateral parietal lobes (involving the posterior cingulate gyrus and the precuneus) detected using 18F-FDG-PET and the Aβ protein and Tau protein deposition detected in the corresponding PET images are regarded as one of the most potential biomarkers for AD (Panegyres et al., 2009; Clark et al., 2012; Mallik et al., 2017; Xia and Dickerson, 2017). MRI data, especially sMRI, mainly provide structural information for diagnosis. Cerebral neurodegenerative structural changes in sMRI, such as a reduction in hippocampal volume and atrophy of some specific cerebral regions (parahippocampal gyrus, amygdala, temporal gyrus, upper parietal lobe, and posterior cingulate gyrus), have been detected in patients with AD (Reitz et al., 2011). The combination of PET and MRI data provides complementary features for AD diagnosis and obtains more promising results than data obtained with a single modality (Mirzaei et al., 2016; Liu et al., 2018). This superiority might be more prominent between two cohorts with small differences, such as patients with pMCI and sMCI. Deep learning methods based on multimodal data have become increasingly popular in diagnosing AD. A GAN can provide Supplementary Data for multimodality studies, as it facilitates the conversion between PET and MRI data. Lin W. et al. (2021) achieved PET and MRI data conversion using a GAN model with reversible blocks. The addition of these blocks improved the non-linear fitting ability of the model and provided images of higher quality. The authors showed high similarity between the generated images of the hippocampal region (as the ROI) and the ground truth (PSNR: 29.34, SSIM: 0.8034 on PET and PSNR: 29.81, SSIM: 0.9389 on MRI). Gao et al. (2021) proposed a GAN model with two pyramid convolution blocks and a self-attention mechanism to achieve MRI-PET data conversion. They also applied the task-induced mechanism in D to preserve important pathological information. The result revealed a high SSIM (0.915 ± 0.04) and PSNR (29.0 ± 2.99) of the generated PET image.

Researchers have also focused on biomarkers detected using different MRI modalities, including sMRI, functional magnetic resonance imaging (fMRI), and diffusion tensor imaging (DTI). For sMRI, alterations in anatomy reflected by T1-weighted MRI, such as atrophy of the hippocampus and rates of brain atrophy, have been extensively investigated (Jack, 2011). In addition, T2 heterogeneity is a potential biomarker reflecting changes in the integrity of brain microstructure and predicting cognitive decline (Wearn et al., 2020). Changes in the microstructure and integrity of white matter are observed on DTI (Sundgren et al., 2004). One study showed that a decrease in fractional anisotropy is detected in multiple posterior white matter regions in patients with AD (Medina et al., 2006). For fMRI, changes in the functional connectivity of different brain regions also have the potential for AD diagnosis (Forouzannezhad et al., 2019). All included studies used T1-weighted MRI data, without GAN based on multimodal MRI data. Except for the potential biomarkers reflected by T1-weighted MRI, those reflected by other MRI modalities are emerging. Although some studies reported the excellent diagnostic performance of multimodal MRI deep learning methods (Hojjati et al., 2018; Marzban et al., 2020), we propose that caution must be exercised in the development of this type of method until these emerging biomarkers are confirmed further.

Some risk factors for AD have been identified, such as the presence of apolipoprotein E (APOE) ε4ε4, depression, diabetes, hypertension, older age, female sex, and lower Mini-Mental State Examination (MMSE) scores (Li et al., 2016; Hersi et al., 2017). Therefore, clinical information may also be considered an important part of multimodal studies. Zhao et al. (2021) considered the function of this information, such as baseline age, sex, education level, and APOE ε4 allele, in the aging simulation process to generate more realistic aging images and obtain accurate predictions for AD progression.

In contrast to the two-stage structure, networks in some studies were established based only on the GAN structure. Baydargil et al. (2021) and Han et al. (2021) only applied a GAN without any other classifiers in anomaly detection for AD, as the D of GAN is actually a classifier. In their study, the G of GAN was run to reconstruct images of subjects based on features learned from images of CN individuals, while the D of GAN was operated to identify patients with AD based on the difference between the reconstructed images and the images of CN individuals. The advantage of this structure over the two-stage structure is that the result of the classification will be fed back to G, ensuring that the generated images have a good classification effect, not simply high quality based on PSNR and SSIM metrics.

Some studies have considered both the two-stage structure and the feedback from the classifier. Zhou X. et al. (2021) applied a GAN to obtain 3-T MRI data from 1.5-T MRI data and further used the generated 3-T imaging data to train an FCN classifier for AD classification. The G of GAN obtained feedback from the FCN and subsequently generate images with good classification effects. They found that the entire diagnostic model exhibited better diagnostic performance.

However, GANs still have some disadvantages when used in practical applications. First, concurrent training of G and D without making a certain network more powerful is a substantial challenge (Sorin et al., 2020). Second, the function of GAN is difficult to interpret. It operates as a black box with visible input and output sides and invisible functions of G and D. The internal logic is difficult to clearly explain.

Our study showed the potential of GAN-based deep learning methods for diagnosing AD and MCI. The following criteria were applied to ensure the diagnosis if possible for the use of this method in clinical practice in the future: (1) diagnoses of other possible brain disorders were excluded; (2) at least one type of neuroimaging data was available, such as sMRI and PET; and (3) the conclusive diagnosis should be based on a combination of the results from GAN methods with those from neuropsychological tests, history analysis, and other clinical diagnoses.

### Limitations and Future Research Directions

Limitations: (1) All included studies used data of AD patients through clinical diagnosis rather than neuropathological examination. Currently, there is still a certain gap between their diagnostic accuracy (Beach et al., 2012). Therefore, the diagnostic performance of GAN methods should be validated further on AD patients diagnosed through neuropathology, even though it is not easy to achieve this goal in the near future. (2) The number of studies included in the meta-analysis of the task of pMCI vs. sMCI classification is relatively small. (3) Due to the limited number of studies, this study only investigated the tasks of AD vs. CN and pMCI vs. sMCI classification. The classification performance of GAN-based deep learning methods must be explored in other tasks, such as the AD vs. MCI vs. CN classification. (3) The lack of subgroup analysis based on the type of data and the method of image processing by GAN is also a limitation of the study.

Some suggestions are provided for future research. First, studies on the task of pMCI vs. sMCI classification and other tasks are needed to further explore the performance of GAN-based deep learning methods. Second, researchers should conduct studies to analyze the roles of the type of data, the type of GAN, and the method of image processing in the diagnostic model. Third, GAN application in other fields (non-medical imaging) may also be considered, such as AD molecular data (Park et al., 2020). Data insufficiency in bioinformatics may be resolved with data augmentation by GAN (Lan et al., 2020). Fourth, using data from patients definitively diagnosed with AD through a neuropathological examination at autopsy rather than a clinical diagnosis would result in methods with more clinical application value.

## Conclusion

This systematic review and meta-analysis reported the good performance of GAN-based deep learning methods in the task of AD vs. CN classification. This good performance is largely attributed to its powerful functions in image processing, including quality improvement, aging simulation, data augmentation, and modality conversion. However, their diagnostic performance in the task of pMCI vs. sMCI classification was not remarkable. Studies using large datasets must be conducted to further explore these methods.

## Data Availability Statement

The original contributions presented in the study are included in the article/Supplementary Material, further inquiries can be directed to the corresponding author/s.

## Author Contributions

CQ, YZ, and TC: conceptualization, methodology, and writing original draft preparation. CQ and YM: software and formal analysis. YZ, QC, YM, HF, and JL: validation. CQ, YZ, YM, QC, JL, ZJ, TC, and QG: writing—review and editing. TC and QG: supervision and funding acquisition. All authors contributed to the article and approved the submitted version.

## Conflict of Interest

The authors declare that the research was conducted in the absence of any commercial or financial relationships that could be construed as a potential conflict of interest.

## Publisher’s Note

All claims expressed in this article are solely those of the authors and do not necessarily represent those of their affiliated organizations, or those of the publisher, the editors and the reviewers. Any product that may be evaluated in this article, or claim that may be made by its manufacturer, is not guaranteed or endorsed by the publisher.
