# Supplementary material for: Diagnostic Performance of Generative Adversarial Network-Based Deep Learning Methods for Alzheimer’s Disease: A Systematic Review and Meta-Analysis
Source: Front Aging Neurosci. 2022 Apr 21;14:841696. doi: 10.3389/fnagi.2022.841696 (PMC9068970; doi:10.3389/fnagi.2022.841696)
Supplement: Supplementary file 1 [file Table_1.docx]

Diagnostic Performance of Generative Adversarial Network-based Deep Learning Methods for Alzheimer’s Disease: A Systematic Review and Meta-Analysis

Supplementary Material

# Supplementary Data

All supplementary files were deposited to FigShare for permanent storage and receive a DOI: 10.6084/m9.figshare.17295887

# Supplementary Tables

Table S1 Search strategy for Pubmed

| Search | Query |
| --- | --- |
| #1 | "Alzheimer Disease"[MeSH Terms] OR "Cognitive Dysfunction"[MeSH Terms] OR "Dementia"[MeSH Terms] |
| #2 | "mild cognitive impairment"[Title/Abstract] OR "MCI"[Title/Abstract] OR "dement*"[Title/Abstract] OR "alzheimer*"[Title/Abstract] OR "AD"[Title/Abstract] |
| #3 | "F-18-FDG"[Title/Abstract] OR "18F-FDG"[Title/Abstract] OR "FDG-PET"[Title/Abstract] OR "Amyloid"[Title/Abstract] OR "Amyloid PET"[Title/Abstract] OR "Tau"[Title/Abstract] OR "Tau-PET"[Title/Abstract] |
| #4 | ("mental*"[All Fields] AND "declin*"[Title/Abstract]) OR "mental*"[All Fields]) AND "impair*"[Title/Abstract]) OR "mental*"[All Fields]) AND "los"[Title/Abstract]) OR "mental*"[All Fields]) AND "deteriorat*"[Title/Abstract]) OR "mental*"[All Fields]) AND "degenerat*"[Title/Abstract]) OR "mental*"[All Fields]) AND "complain*"[Title/Abstract]) OR "mental*"[All Fields]) AND "disturb*"[Title/Abstract]) OR "mental*"[All Fields]) AND "disorder*"[Title/Abstract]) |
| #5 | ("cerebr*"[All Fields] AND "declin*"[Title/Abstract]) OR "cerebr*"[All Fields]) AND "impair*"[Title/Abstract]) OR "cerebr*"[All Fields]) AND "los"[Title/Abstract]) OR "cerebr*"[All Fields]) AND "deteriorat*"[Title/Abstract]) OR "cerebr*"[All Fields]) AND "degenerat*"[Title/Abstract]) OR "cerebr*"[All Fields]) AND "complain*"[Title/Abstract]) OR "cerebr*"[All Fields]) AND "disturb*"[Title/Abstract]) OR "cerebr*"[All Fields]) AND "disorder*"[Title/Abstract]) |
| #6 | ("memory declin*"[Title/Abstract] OR "memory impair*"[Title/Abstract] OR "memory los*"[Title/Abstract] OR "memory deteriorat*"[Title/Abstract] OR "memory degenerat*"[Title/Abstract] OR "memory complain*"[Title/Abstract] OR "memory disturb*"[Title/Abstract] OR "memory disorder*"[Title/Abstract]) |
| #7 | ("cognit*"[All Fields] AND "declin*"[Title/Abstract]) OR "cognit*"[All Fields]) AND "impair*"[Title/Abstract]) OR "cognit*"[All Fields]) AND "los"[Title/Abstract]) OR "cognit*"[All Fields]) AND "deteriorat*"[Title/Abstract]) OR "cognit*"[All Fields]) AND "degenerat*"[Title/Abstract]) OR "cognit*"[All Fields]) AND "complain*"[Title/Abstract]) OR "cognit*"[All Fields]) AND "disturb*"[Title/Abstract]) OR "cognit*"[All Fields]) AND "disorder*"[Title/Abstract])) |
| #8 | #1 OR #2 OR #3 OR #4 OR #5 OR #6 OR #7 |
| #9 | "generative model*"[Title/Abstract] OR "adversarial network*"[Title/Abstract] OR "generative adversarial network*"[Title/Abstract] |
| #10 | #8 AND #9 |

Table S2 Search strategy for EMBASE

| Search | Query |
| --- | --- |
| #1 | 'alzheimer disease'/exp OR 'dementia'/exp OR 'mild cognitive impairment'/exp |
| #2 | alzheimer*:ab,ti OR 'ad':ab,ti OR dement*:ab,ti OR 'mild cognitive impairment':ab,ti OR 'mci':ab,ti |
| #3 | 'f-18-fdg':ab,ti OR '18f-fdg':ab,ti OR 'fdg-pet':ab,ti OR 'amyloid':ab,ti OR 'amyloid pet':ab,ti OR 'tau':ab,ti OR 'tau-pet':ab,ti |
| #4 | 'mental* declin*':ab,ti OR 'mental* impair*':ab,ti OR 'mental* los*':ab,ti OR 'mental* deteriorat*':ab,ti OR 'mental* degenerat*':ab,ti OR 'mental* complain*':ab,ti OR 'mental* disturb*':ab,ti OR 'mental* disorder*':ab,ti |
| #5 | 'cerebr* declin*':ab,ti OR 'cerebr* impair*':ab,ti OR 'cerebr* los*':ab,ti OR 'cerebr* deteriorat*':ab,ti OR 'cerebr* degenerat*':ab,ti OR 'cerebr* complain*':ab,ti OR 'cerebr* disturb*':ab,ti OR 'cerebr* disorder*':ab,ti |
| #6 | 'memory declin*':ab,ti OR 'memory impair*':ab,ti OR 'memory los*':ab,ti OR 'memory deteriorat*':ab,ti OR 'memory degenerat*':ab,ti OR 'memory complain*':ab,ti OR 'memory disturb*':ab,ti OR 'memory disorder*':ab,ti |
| #7 | 'cognit* declin*':ab,ti OR 'cognit* impair*':ab,ti OR 'cognit* los*':ab,ti OR 'cognit* deteriorat*':ab,ti OR 'cognit* degenerat*':ab,ti OR 'cognit* complain*':ab,ti OR 'cognit* disturb*':ab,ti OR 'cognit* disorder*':ab,ti |
| #8 | #1 OR #2 OR #3 OR #4 OR #5 OR #6 OR #7 |
| #9 | 'adversarial network*':ab,ti OR 'generative adversarial network*':ab,ti) OR 'generative adversarial network'/exp) OR 'generative model*':ab,ti |
| #10 | #8 AND #9 |

Table S3 Search strategy for Cochrane Library

| Search | Query |
| --- | --- |
| #1 | "Alzheimer Disease"[MeSH Terms] OR "Cognitive Dysfunction"[MeSH Terms] OR "Dementia"[MeSH Terms] |
| #2 | ("AD"):ti,ab,kw OR (Alzheimer*):ti,ab,kw OR (dement*):ti,ab,kw OR ("mild cognitive impairment"):ti,ab,kw OR ("MCI"):ti,ab,kw |
| #3 | ("F-18-FDG"):ti,ab,kw OR ("18F-FDG"):ti,ab,kw OR ("FDG-PET"):ti,ab,kw OR ("Amyloid"):ti,ab,kw OR ("Amyloid PET"):ti,ab,kw OR ("Tau"):ti,ab,kw OR ("Tau-PET"):ti,ab,kw |
| #4 | (mental* NEXT declin*):ti,ab,kw OR (mental* NEXT impair*):ti,ab,kw OR (mental* NEXT los*):ti,ab,kw OR (mental* NEXT deteriorat*):ti,ab,kw OR (mental* NEXT degenerat*):ti,ab,kw OR (mental* NEXT complain*):ti,ab,kw OR (mental* NEXT disturb*):ti,ab,kw OR (mental* NEXT disorder*):ti,ab,kw |
| #5 | (cerebr* NEXT declin*):ti,ab,kw OR (cerebr* NEXT impair*):ti,ab,kw OR (cerebr* NEXT los*):ti,ab,kw OR (cerebr* NEXT deteriorat*):ti,ab,kw OR (cerebr* NEXT degenerat*):ti,ab,kw OR (cerebr* NEXT complain*):ti,ab,kw OR (cerebr* NEXT disturb*):ti,ab,kw OR (cerebr* NEXT disorder*):ti,ab,kw |
| #6 | (memory NEXT declin*):ti,ab,kw OR (memory NEXT impair*):ti,ab,kw OR (memory NEXT los*):ti,ab,kw OR (memory NEXT deteriorat*):ti,ab,kw OR (memory NEXT degenerat*):ti,ab,kw OR (memory NEXT complain*):ti,ab,kw OR (memory NEXT disturb*):ti,ab,kw OR (memory NEXT disorder*):ti,ab,kw |
| #7 | (cognit* NEXT declin*):ti,ab,kw OR (cognit* NEXT impair*):ti,ab,kw OR (cognit* NEXT los*):ti,ab,kw OR (cognit* NEXT deteriorat*):ti,ab,kw OR (cognit* NEXT degenerat*):ti,ab,kw OR (cognit* NEXT complain*):ti,ab,kw OR (cognit* NEXT disturb*):ti,ab,kw OR (cognit* NEXT disorder*):ti,ab,kw |
| #8 | #1 OR #2 OR #3 OR #4 OR #5 OR #6 OR #7 |
| #9 | ("generative adversarial network"):ti,ab,kw OR ("adversarial network"):ti,ab,kw OR ("adversarial networks"):ti,ab,kw OR ("generative adversarial networks"):ti,ab,kw OR ("generative model"):ti,ab,kw OR ("generative models"):ti,ab,kw |
| #10 | #8 AND #9 |

Table S4 Search strategy for Web of Science

| Search | Query |
| --- | --- |
| #1 | TS=("Alzheimer*") OR TS=("AD") OR TS=("dement*") OR TS=("mild cognitive impairment") OR TS=("MCI") |
| #2 | TS=("F-18-FDG") OR TS=("18F-FDG") OR TS=("FDG-PET") OR TS=("Amyloid") OR TS=("Amyloid PET") OR TS=("Tau") OR TS=("Tau-PET") |
| #3 | TS=("mental* declin*") OR TS=("mental* impair*") OR TS=("mental* los*") OR TS=("mental* deteriorat*") OR TS=("mental* degenerat*") OR TS=("mental* complain*") OR TS=("mental* disturb*") OR TS=("mental* disorder*") |
| #4 | TS=("cerebr* declin*") OR TS=("cerebr* impair*") OR TS=("cerebr* los*") OR TS=("cerebr* deteriorat*") OR TS=("cerebr* degenerat*") OR TS=("cerebr* complain*") OR TS=("cerebr* disturb*") OR TS=("cerebr* disorder*") |
| #5 | TS=("memory declin*") OR TS=("memory impair*") OR TS=("memory los*") OR TS=("memory deteriorat*") OR TS=("memory degenerat*") OR TS=("memory complain*") OR TS=("memory disturb*") OR TS=("memory disorder*") |
| #6 | TS=("cognit* declin*") OR TS=("cognit* impair*") OR TS=("cognit* los*") OR TS=("cognit* deteriorat*") OR TS=("cognit* degenerat*") OR TS=("cognit* complain*") OR TS=("cognit* disturb*") OR TS=("cognit* disorder*") |
| #7 | #1 OR #2 OR #3 OR #4 OR #5 OR #6 |
| #8 | TS=("adversarial network*") OR TS=("generative adversarial network*") OR TS=("generative model*") |
| #9 | #7 AND #8 |

| **Section and Topic** | **Item #** | **Checklist item** | **Location where item is reported** |
| --- | --- | --- | --- |
| **TITLE** | | |  |
| Title | 1 | Identify the report as a systematic review. | Title |
| **ABSTRACT** | | |  |
| Abstract | 2 | See the PRISMA 2020 for Abstracts checklist. | Abstract |
| **INTRODUCTION** | | |  |
| Rationale | 3 | Describe the rationale for the review in the context of existing knowledge. | Introduction |
| Objectives | 4 | Provide an explicit statement of the objective(s) or question(s) the review addresses. | Introduction |
| **METHODS** | | |  |
| Eligibility criteria | 5 | Specify the inclusion and exclusion criteria for the review and how studies were grouped for the syntheses. | Materials and Methods, Inclusion and exclusion criteria |
| Information sources | 6 | Specify all databases, registers, websites, organisations, reference lists and other sources searched or consulted to identify studies. Specify the date when each source was last searched or consulted. | Materials and Methods, Literature search |
| Search strategy | 7 | Present the full search strategies for all databases, registers and websites, including any filters and limits used. | Supplementary material, Table S1; Table S2; Table S3; Table S4 |
| Selection process | 8 | Specify the methods used to decide whether a study met the inclusion criteria of the review, including how many reviewers screened each record and each report retrieved, whether they worked independently, and if applicable, details of automation tools used in the process. | Materials and Methods, Article screening |
| Data collection process | 9 | Specify the methods used to collect data from reports, including how many reviewers collected data from each report, whether they worked independently, any processes for obtaining or confirming data from study investigators, and if applicable, details of automation tools used in the process. | Materials and Methods, Data extraction |
| Data items | 10a | List and define all outcomes for which data were sought. Specify whether all results that were compatible with each outcome domain in each study were sought (e.g. for all measures, time points, analyses), and if not, the methods used to decide which results to collect. | Materials and Methods, Data analysis |
|  | 10b | List and define all other variables for which data were sought (e.g. participant and intervention characteristics, funding sources). Describe any assumptions made about any missing or unclear information. | Materials and Methods, Data extraction |
| Study risk of bias assessment | 11 | Specify the methods used to assess risk of bias in the included studies, including details of the tool(s) used, how many reviewers assessed each study and whether they worked independently, and if applicable, details of automation tools used in the process. | Materials and Methods, Quality Assessment |
| Effect measures | 12 | Specify for each outcome the effect measure(s) (e.g. risk ratio, mean difference) used in the synthesis or presentation of results. | Materials and Methods, Data analysis |
| Synthesis methods | 13a | Describe the processes used to decide which studies were eligible for each synthesis (e.g. tabulating the study intervention characteristics and comparing against the planned groups for each synthesis (item #5)). | None |
|  | 13b | Describe any methods required to prepare the data for presentation or synthesis, such as handling of missing summary statistics, or data conversions. | Materials and Methods, Data analysis |
|  | 13c | Describe any methods used to tabulate or visually display results of individual studies and syntheses. | Materials and Methods, Data analysis |
|  | 13d | Describe any methods used to synthesize results and provide a rationale for the choice(s). If meta-analysis was performed, describe the model(s), method(s) to identify the presence and extent of statistical heterogeneity, and software package(s) used. | Materials and Methods, Data analysis |
|  | 13e | Describe any methods used to explore possible causes of heterogeneity among study results (e.g. subgroup analysis, meta-regression). | None |
|  | 13f | Describe any sensitivity analyses conducted to assess robustness of the synthesized results. | None |
| Reporting bias assessment | 14 | Describe any methods used to assess risk of bias due to missing results in a synthesis (arising from reporting biases). | None |
| Certainty assessment | 15 | Describe any methods used to assess certainty (or confidence) in the body of evidence for an outcome. | None |
| **RESULTS** | | |  |
| Study selection | 16a | Describe the results of the search and selection process, from the number of records identified in the search to the number of studies included in the review, ideally using a flow diagram. | Results, Study selection |
|  | 16b | Cite studies that might appear to meet the inclusion criteria, but which were excluded, and explain why they were excluded. | Figure 1 |
| Study characteristics | 17 | Cite each included study and present its characteristics. | Results, Characteristics of the included studies |
| Risk of bias in studies | 18 | Present assessments of risk of bias for each included study. | Results, Quality assessment |
| Results of individual studies | 19 | For all outcomes, present, for each study: (a) summary statistics for each group (where appropriate) and (b) an effect estimate and its precision (e.g. confidence/credible interval), ideally using structured tables or plots. | Table 1 |
| Results of syntheses | 20a | For each synthesis, briefly summarise the characteristics and risk of bias among contributing studies. | Results, Diagnostic performance of generative adversarial network-based deep learning methods |
|  | 20b | Present results of all statistical syntheses conducted. If meta-analysis was done, present for each the summary estimate and its precision (e.g. confidence/credible interval) and measures of statistical heterogeneity. If comparing groups, describe the direction of the effect. | Results, Diagnostic performance of generative adversarial network-based deep learning methods |
|  | 20c | Present results of all investigations of possible causes of heterogeneity among study results. | None |
|  | 20d | Present results of all sensitivity analyses conducted to assess the robustness of the synthesized results. | None |
| Reporting biases | 21 | Present assessments of risk of bias due to missing results (arising from reporting biases) for each synthesis assessed. | None |
| Certainty of evidence | 22 | Present assessments of certainty (or confidence) in the body of evidence for each outcome assessed. | None |
| **DISCUSSION** | | |  |
| Discussion | 23a | Provide a general interpretation of the results in the context of other evidence. | Discussion, The function of generative adversarial network in the diagnostic model |
|  | 23b | Discuss any limitations of the evidence included in the review. | Discussion, Limitations and future research direction |
|  | 23c | Discuss any limitations of the review processes used. | Discussion, Limitations and future research direction |
|  | 23d | Discuss implications of the results for practice, policy, and future research. | Discussion, Limitations and future research direction |
| **OTHER INFORMATION** | | |  |
| Registration and protocol | 24a | Provide registration information for the review, including register name and registration number, or state that the review was not registered. | Materials and Methods, Protocol and registration |
|  | 24b | Indicate where the review protocol can be accessed, or state that a protocol was not prepared. | Materials and Methods, Protocol and registration |
|  | 24c | Describe and explain any amendments to information provided at registration or in the protocol. | None |
| Support | 25 | Describe sources of financial or non-financial support for the review, and the role of the funders or sponsors in the review. | Funding |
| Competing interests | 26 | Declare any competing interests of review authors. | Conflicts of Interest |
| Availability of data, code and other materials | 27 | Report which of the following are publicly available and where they can be found: template data collection forms; data extracted from included studies; data used for all analyses; analytic code; any other materials used in the review. | None |

*From:*  Page MJ, McKenzie JE, Bossuyt PM, Boutron I, Hoffmann TC, Mulrow CD, et al. The PRISMA 2020 statement: an updated guideline for reporting systematic reviews. BMJ 2021;372:n71. doi: 10.1136/bmj.n71

For more information, visit: <http://www.prisma-statement.org/>
